# Facile In-Situ Fabrication of a Ternary ZnO/TiO_2_/Ag Nanocomposite for Enhanced Bactericidal and Biocompatibility Properties

**DOI:** 10.3390/antibiotics10010086

**Published:** 2021-01-18

**Authors:** Priyadarshini Sakthi Mohan, Faridah Sonsuddin, Azizah Binti Mainal, Rosiyah Yahya, Gopinath Venkatraman, Jamuna Vadivelu, Dunia A. Al-Farraj, Amal M. Al-Mohaimeed, Khaloud Mohammed Alarijani

**Affiliations:** 1 Department of Chemistry, Faculty of Science, University of Malaya, Kuala Lumpur 50603, Malaysia; priyadarshini88@gmail.com (P.S.M.); azizah_mainal@um.edu.my (A.B.M.); 2 Centre for Foundation Studies in Science, University of Malaya, Kuala Lumpur 50603, Malaysia; sfaridah@um.edu.my; 3 Department of Medical Microbiology, Faculty of Medicine, University of Malaya, Kuala Lumpur 50603, Malaysia; yele.gopi@gmail.com (G.V.); jamuna@ummc.edu.my (J.V.); 4 Department of Botany and Microbiology, College of Science, King Saud University, Riyadh 11451, Saudi Arabia; dfarraj@ksu.edu.sa (D.A.A.-F.); kalarjani@ksu.edu.sa (K.M.A.); 5 Department of Chemistry, College of Science, King Saud University, P.O. Box 22452, Riyadh 11495, Saudi Arabia; muhemeed@ksu.edu.sa

**Keywords:** antibacterial, semiconductor materials, nanocomposites, precipitation, cytotoxicity

## Abstract

This paper presents for the first time a successful fabrication of ternary ZnO/TiO_2_/Ag nanocomposites consisting of zinc oxide (ZnO), titania (TiO_2_) and silver (Ag) nanoparticles (NPs) synthesised using *Morinda citrifolia* fruit (MCF) extract. ZnONPs were synthesised using the co-precipitation method, and TiO_2_ and Ag were introduced into the precursor solutions under microwave irradiation to obtain ZnO/TiO_2_/Ag nanocomposites (NCs). This material demonstrated enhanced bactericidal effect towards bacterial pathogens compared to that of the binary TiO_2_/Ag, Ag and TiO_2_ alone. In vitro cytotoxicity results of the as-synthesised ZnO/TiO_2_/AgNCs on RAW 264.7 macrophages and A549 cell lines revealed a negative role in cytotoxicity, but contributed astoundingly towards antimicrobials as compared of Ag alone and binary Ag/TiO_2_. This study shows that the resultant ternary metal/bi-semiconductor nanocomposites may provide a therapeutic strategy for the eradication of bacterial pathogens without affecting the healthy mammalian cells.

## 1. Introduction

Nanomaterials have attracted much attention in biopharmaceutical industries due to their unique physicochemical and functional characteristics. Among metal oxide nanostructure semiconductors, ZnO and TiO_2_ have drawn significant interest because of their non-toxic nature and easily tunable properties [1,2]. To date, progress in nanotechnology provides novel functional materials by tailoring semiconductor nanostructures with suitable metal nanoparticles. In another aspect, the rapid increase in antimicrobial resistance and ineffectiveness of drugs have resulted in a search of emerging alternate antimicrobials with improved biomedical properties [3]. Thus, the race to find alternate antimicrobials over multidrug-resistant pathogens using nanotechnological tools is a hotspot among researchers [4]. It has been reported that the nanomaterials demonstrated better bactericidal activity over existing antibiotics and other antimicrobial materials [5,6].

The known antimicrobial effect of metal and metal oxide nanoparticles is mainly due to their nano-size, large surface-to-volume ratio and high surface reactivity that enables close interactions with the microbial communities [7]. Among the nanostructured materials, Ag is considered as a superior antimicrobial due to their strong interaction with the bacterial surface with the release of Ag^+^ ions, which will damage the cell membrane structure and modulating the cell signalling pathways [8,9]. However, Ag nanoparticles (AgNPs) acute toxicity has been reported towards the human cells is the rising concerns in its biomedical functionalities. Taking into account, the AgNPs toxicity was significantly reduced by incorporating it with semiconductor materials [10].

ZnO and TiO_2_ have unique physical and optoelectrical properties because of their dual semiconducting and piezoelectric properties with a wide bandgap of 3.37 eV and 3.2 eV, respectively [11]. These materials are categorised under Generally Recognised As Safe (GRAS) materials for human consumption by the United States Food and Drugs Administration (USFDA) [12,13]. Owing to their remarkable properties, these materials have been widely used as potential antibacterial, anticancer, catalysis, sensor, optic, optoelectronic and energy materials [14]. Semiconductors TiO_2_ and ZnO attract light energy in the UV range because of their large bandgap and high stability, rendering them suitable for UV screening applications [15]. The bactericidal mechanism of ZnO or TiO_2_ NPs have been suggested due to the generation of reactive oxygen species (ROS) on the bacterial cell membrane, altering the metabolic functions and thus leads to the bacterial cell inactivation [16]. It was also suggested that the nanoparticles could have undergone internalisation through the cell membrane damage, which could efficiently kill the bacterial pathogens [17,18]. Based on the literature, ZnONPs bacterial toxicity also depends on their structural morphology and surface charge [5].

Physical and chemical synthesis techniques are widely applied for the preparation of metal and metal oxide nanoparticles. However, these methods involve the use of expensive instrumentation, high energy consumption and hazardous chemicals, resulting in severe health defects to humans and the environment [19]. To overcome these issues, biosynthesis has been widely applied in recent decades for its eco-friendly synthesis procedures [20]. Compared to conventional heating, microwave irradiation is a promising method because of the homogenous heating, energy efficiency and rapid rate of nanoparticle formation in conjugation with the plant extract [21]. Therefore, the microwave assisted heating is a simple, rapid and low-cost method for synthesising nanomaterials and is suitable for large-scale production [22]. Metal-semiconductor nanocomposites combine the properties of individual components to deliver their synergistic effect, which has exciting applications. Thus, they behave completely different from the component materials making the research very interesting [15]. To the best of our knowledge, nanocomposites of such type using *Morinda citrifolia* fruits have not been reported before. The medicinal plant *M. citrifolia*, which belongs to the family Rubiaceae and is commonly called noni or Indian mulberry, is widely present in the Asian tropical countries. It has been used to treat various ailments, such as hypertension, diabetes and chronic diseases, and has also been used as a food supplement [23]. In this study, the synthesised nanomaterials could be used as an excellent antimicrobial, as these materials have outstanding antimicrobial properties with minimal cytotoxicity.

## 2. Results and Discussion

### 2.1. Bioanalytical Characterisation of the Synthesised Nanomaterials

#### 2.1.1. UV-Vis Analysis

**Figure 1:**
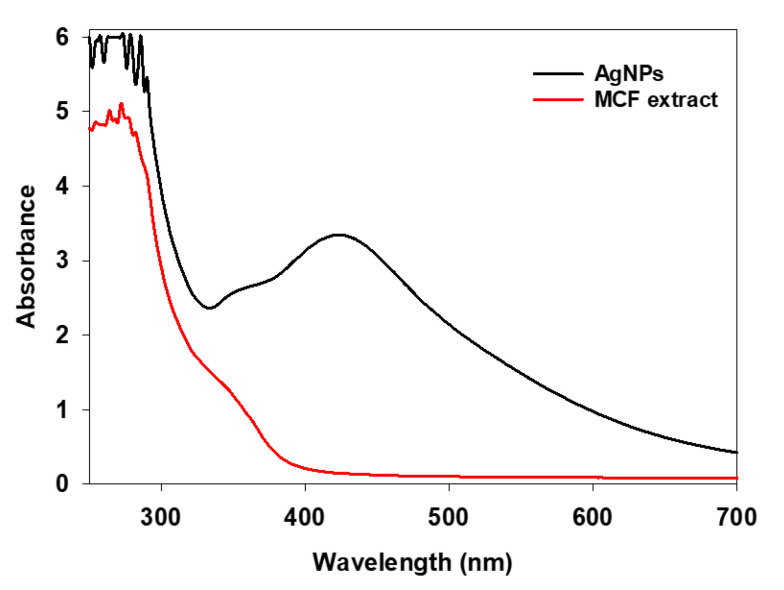
UV-Vis absorption spectra of *Morinda citrifolia* fruit (MCF) extract and microwave mediated Ag nanoparticle (AgNP) solution.

The metallic nanoparticles formation in aqueous medium was generally characterised using UV-vis spectroscopy. The UV-vis spectrum of MCF mediated resultant brown colour solution (Figure 1) showed a prominent peak around 420 nm, which is the characteristic peak of AgNPs. The result is in accordance with the earlier findings [24]. The observed brown colour is due to the surface plasmon resonance of metallic silver, which is in concord with the light source [25]. The UV-vis spectrum of fruit extract alone showed a broad peak at 280 nm, which is characteristics of active biomolecules in the MCF. The active functional biomolecules present in the MCF extract triggers the nanoparticles synthesis, whereby the phytochemical compounds attract the silver salt precursor via electrostatic interactions and promote the bioreduction of Ag^+^ ions, resulting in the nucleation of silver atoms. Furthermore, the growth of the nanoparticles was achieved by the amalgamation of subsequently reduced silver nuclei. It has been reported that almost 200 biomolecules were characterised from the *M. citrifolia,* which are the reason for their biological functions [26]. Moreover, a recent report described that the phytochemicals ascorbic acid, flavonoid, caproic acid and quercetin, present in the MCF aqueous extract, are involved in silver ions reduction [27].

#### 2.1.2. Detection of the Functional Group Responsible for NPs Synthesis

**Figure 2:**
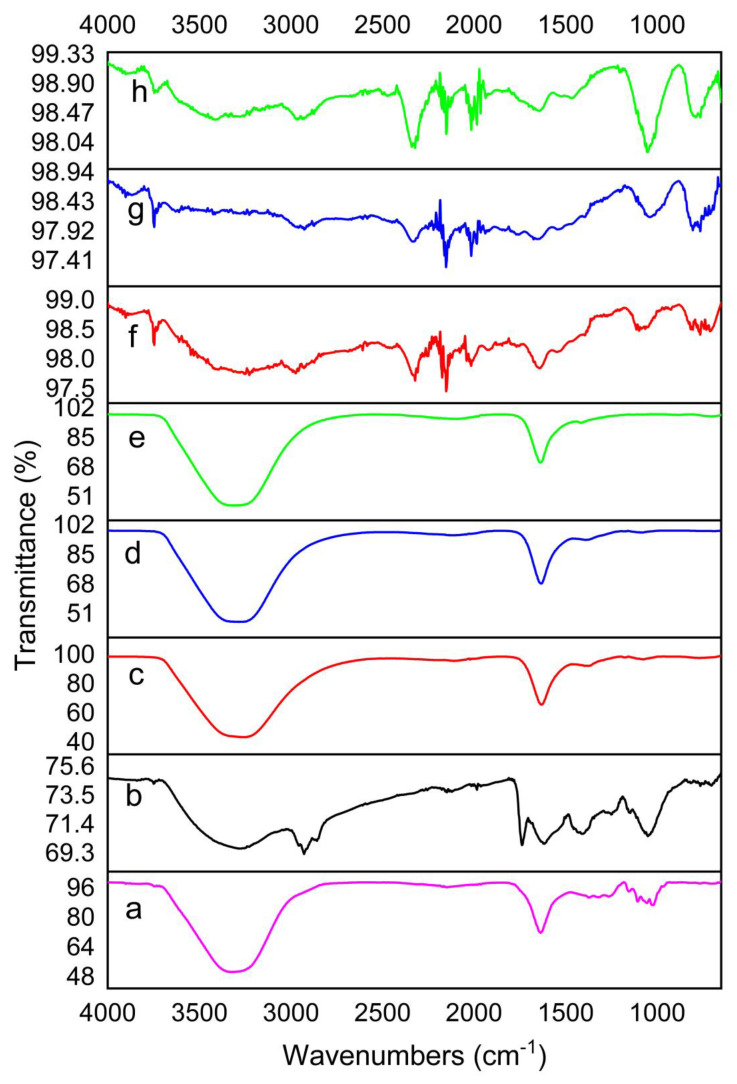
FTIR spectra of (**a**) MCF alone, (**b**) synthesised AgNPs, (**c**), microwave-synthesised TiO_2_NPs (**d**) TiO_2_/AgNCs, (**e**) ZnO/TiO_2_/AgNCs and (**f**) calcined TiO_2_NPs, (**g**) TiO_2_/AgNCs and (**h**) ZnO/TiO_2_/AgNCs.

The FTIR spectra of MCF alone and the four different NPs synthesised (AgNPs, TiO_2_NPs, TiO_2_/AgNCs and ZnO/TiO_2_/AgNCs) are shown in Figure 2. MCF extract exhibited peaks at 3326, 2148, 1635, 1314, 1107 and 1020 cm^−1^, which are attributed to hydroxyl, stretching alkyne bond, amide I, CH_2_ scissoring vibration, C-O and C-O-C [28,29,30]. AgNPs displayed peaks at 3278, 1738, 1615, 1410 and 1050 cm^−1^, while the microwave-synthesised TiO_2_NPs showed five intense peaks at 3257, 2109, 1633 and 1397 cm^−1^. As for TiO_2_/AgNCs and ZnO/TiO_2_/AgNCs precipitates, peaks are observed at 3284, 2121, 1634 and 1083 and 3308, 2090 and 1636 cm^−1^, respectively. Compared to the FTIR spectra of MCF and microwave-synthesised TiO_2_NPs, TiO_2_/AgNCs and ZnO/TiO_2_/AgNCs, major shift changes are observed from the hydroxyl, stretching alkynes bond, carbonyl and CH_2_ vibrations. These results support the earlier findings that bioactive functional groups could have strongly interacted with the metal ions to reduce the particle to nanosize [31]. In the case of calcined TiO_2_NPs, TiO_2_/AgNCs and ZnO/TiO_2_/AgNCs, there was no significant peak detected, due to the degradation of bioactive molecules during the calcination process.

#### 2.1.3. XRD Analysis

**Figure 3:**
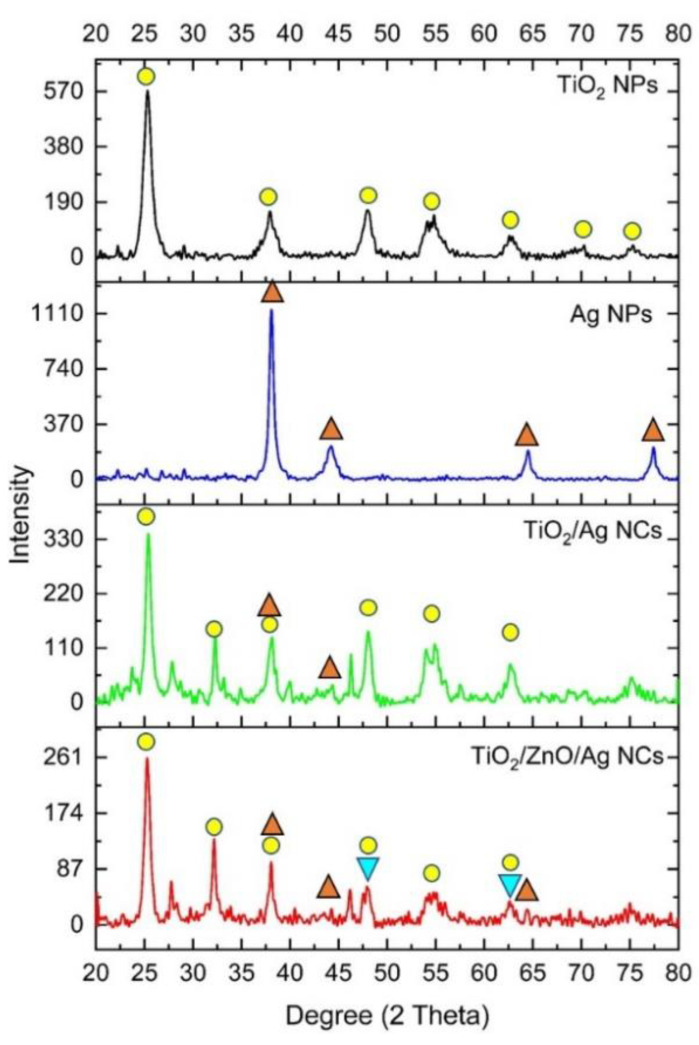
XRD pattern of TiO_2_NPs, AgNPs, TiO_2_/AgNCs and ZnO/TiO_2_/AgNCs.

XRD studies of four of the different nanoparticles (AgNPs, TiO_2_NPs, TiO_2_/AgNCs and ZnO/TiO_2_/AgNCs) derived from MCF are shown in (Figure 3). The XRD pattern of AgNPs showed peaks at 38.0°, 44.1°, 64.4° and 77.3° corresponding to crystal planes (hkl) of 111, 200, 220 and 311, respectively. The resultant data were matched with ICSD NO. 98-060-4629, which represent the face centred cubic structure of AgNPs. TiO_2_NPs exhibited seven peaks at 25.3°, 37.9°, 48.2°, 54.8°, 62.8°, 70.3° and 75.2°, corresponding to the anatase phase of TiO_2_NPs, which matched with the ICSD no: 98-015-4604. The diffraction pattern of TiO_2_NPs doped AgNPs exhibited the additional Ag peaks at 38.0°, 44.1°, 64.4° and 77.3° along with the TiO_2_NPs corresponding to the lattice planes of Ag and TiO_2_NPs anatase nanocrystallites, respectively, confirming the formation of TiO_2_/AgNCs. Furthermore, ZnO modification on the TiO_2_/Ag nanocomposite exhibited additional peaks at 31.8°. 47.7°, 62.8° and 67.0° attributed to the characteristics of ZnO nanocrystallites, which matched the ICSD file no. 98-016-5011, which approves the materialisation of ZnO/TiO_2_/AgNCs formation.

#### 2.1.4. FESEM, EDX and Elemental Mapping

**Figure 4:**
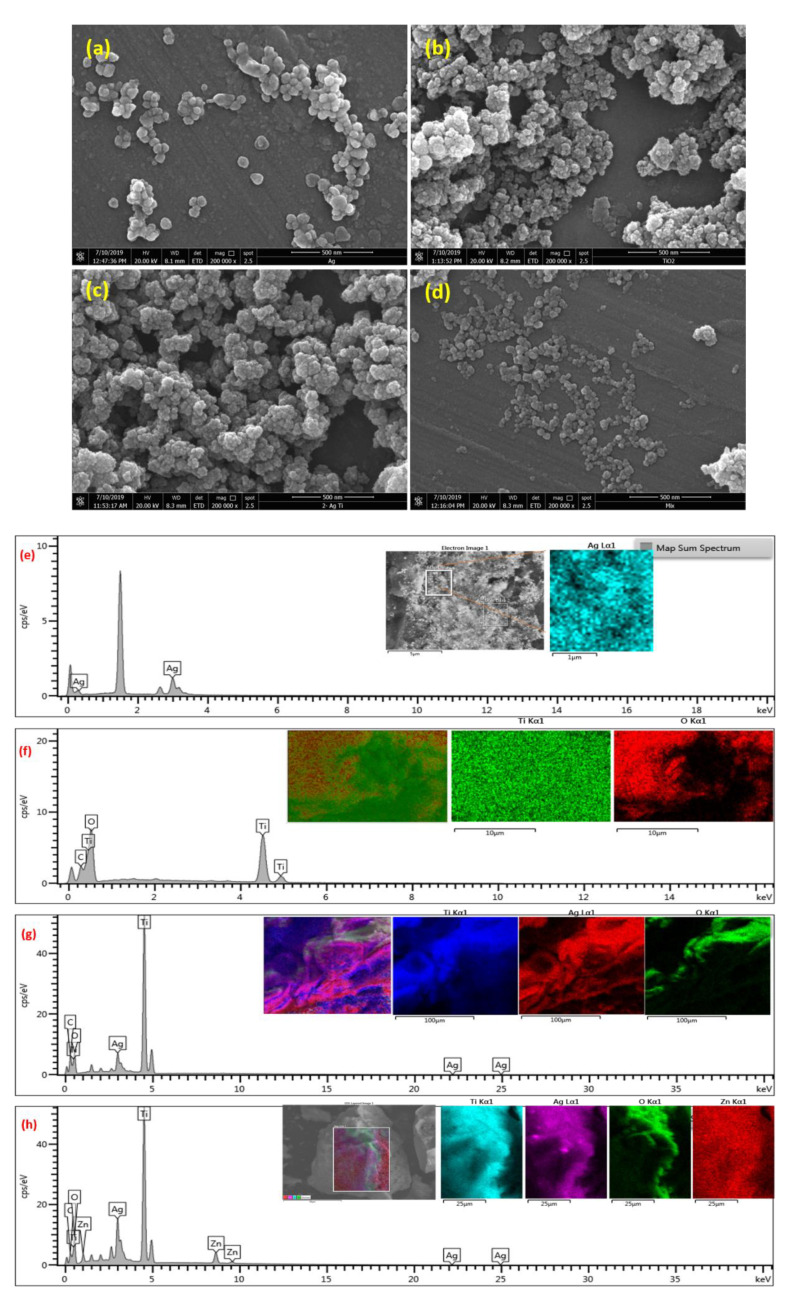
FESEM images of AgNPs (**a**), TiO_2_NPs (**b**), TiO_2_/AgNCs (**c**) and ZnO/TiO_2_/AgNCs (**d**). EDX analysis (Elemental mapping + Elemental composition) detection of AgNPs (**e**) TiO_2_NPs, (**f**) TiO_2_/AgNCs, (**g**) and ZnO/TiO_2_/AgNCs (**h**).

The FESEM images of TiO_2_NPs showed these particles are spherical shaped with an average size of 22 nm (Figure 4a). In the case of AgNPs, two different shapes of nanoparticles (spherical and triangular) were observed with an average size of 35 nm (Figure 4b). The hybrid combination of TiO_2_/AgNCs exhibited almost spherical shaped particles with an average size of 26 nm (Figure 4c). ZnO/TiO_2_/AgNCs samples showed anisotropic structured particles (spherical and irregular) at an average size of 40 nm (Figure 4d).

EDX compositional analysis of the resultant nanoparticles displayed elemental signals of Ag and Ti, O for AgNPs and TiO_2_NPs, respectively (Figure 4e,f). In the case of TiO_2_/AgNCs and ZnO/TiO_2_/AgNCs, the elemental compositions are confirmed by the existence of Ti, O and Ag and Ti, Zn, O and Ag signals, respectively (Figure 4g,h). In addition, the elemental mapping results revealed further the distribution of elements (Ti, Zn, O and Ag) on the surface of the nanoparticle with different colour observation.

#### 2.1.5. Zeta Potential Surface Charge Analysis

**Figure 5:**
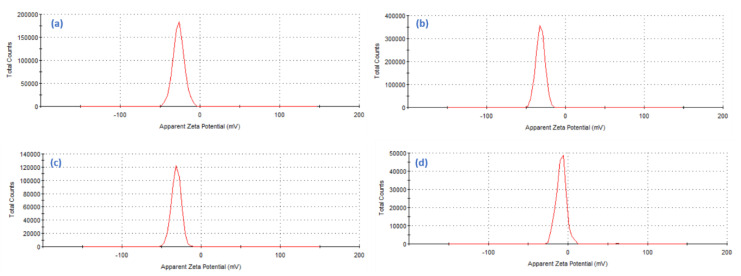
Zeta potential surface charge of (**a**) AgNPs, (**b**) TiO_2_NPs, (**c**) TiO_2_/AgNCs and (**d**) ZnO/TiO_2_/AgNCs.

Assessment of resultant colloidal nanoparticles surface charges via zeta potential analysis is the key factor to determine their stability. The zeta potentials of the AgNPs, TiO_2_NPs, TiO_2_/AgNCs and ZnO/TiO_2_/AgNCs were measured, and it is evident to have a highly negative charge with good colloidal stability because of its repulsive forces, which prevent the agglomeration of the nanoparticles. Values of the zeta potential of AgNPs and TiO_2_NPs at −26.9 mV and −32.0 mV, respectively, indicate that both nanoparticles were negatively charged (Figure 5a,b). However, the zeta potential values of TiO_2_/AgNCs expressed the zeta values of −31.6 mV and the zeta values of ZnO/TiO_2_/AgNCs was comparatively lesser at −7.3 mV (Figure 5c,d). This corresponds to weaker stability of nanocomposites due to the addition of metal and semiconductor materials.

### 2.2. Antimicrobial Properties

#### 2.2.1. Minimum Inhibitory Concentration (MIC)

The antibacterial activities of AgNPs, TiO_2_NPs, TiO_2_/AgNCs and ZnO/TiO_2_/AgNCs were tested against the nosocomial human bacterial pathogens. MIC results revealed that the TiO_2_NPs alone did not show any significant reduction on bacterial growth. However, at a higher dosage, TiO_2_NPs inhibited the growth of *B. subtilis* at a concentration of 250 μg/mL and *E. coli*, *S. aureus, P. aeruginosa* at 500 μg/mL, respectively. To enhance the bioactivity of TiO_2_NPs, it was fabricated with the Ag and ZnO/Ag materials where it displayed significant bactericidal activity as confirmed by MIC assay results (Table 1). It is also noted that the bacterial inhibitory effect of AgNPs and TiO_2_/AgNCs were obvious, but in the case of ZnO/TiO_2_/AgNCs, it was not reported yet. From the results, MIC of AgNPs was found to be 31.2 and 62.5 μg/mL for *E. coil* and *B. subtilis* and *S. aureus* and *P. aeruginosa*, respectively. In contrary, the TiO_2_/AgNCs MIC was found to be 31.2 μg/mL for *E. coil, B. subtilis* and *P. aeruginosa* and 62.5 μg/mL for *S. aureus*. In the case of ZnO/TiO_2_/AgNCs treatment with bacteria, it demonstrated higher inhibition at a concentration of 31.2 μg/mL and 15.6 μg/mL for *E. coil* and *S. aureus* and *P. aeruginosa* and *B. subtilis*. Green synthesis of AgNPs demonstrated superior bactericidal activity due to its membrane penetration and release of silver ions (Ag^+^), which interact with the thiol group of a bacterial enzyme, interrupting the respiratory mechanism by having a lethal effect on the bacteria [32]. In addition, the synergistic effect could be observed due to various mechanisms, including Ti^4+^ or Zn^2+^ ions release from the respective nanocomposite formulation, which enables higher production of reactive oxygen species (ROS) on the surfaces of the nanoparticles, which facilitates the oxidative stress or devastate the bacterial defence mechanism [33]. Based on the above-stated reason, the resultant ZnO/TiO_2_/AgNCs demonstrated a remarkable bactericidal effect compared to the material alone or TiO_2_/Ag nanocomposites.

#### 2.2.2. Bacterial Metabolic Activity

**Figure 6:**
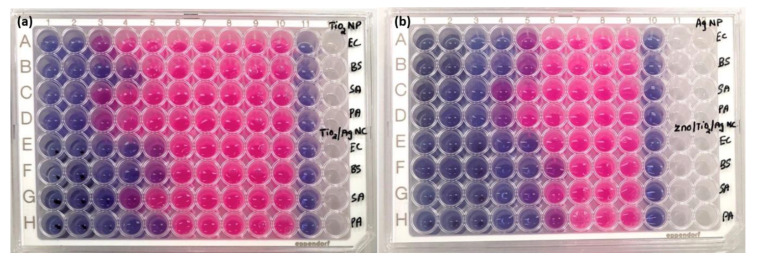
(**a**) Metabolic changes of bacterial pathogens of *E. coli, B. subtilis, S. aureus* and *P. aeruginosa* caused by TiO_2_NPs (rows A–D) and TiO_2_/AgNCs (rows E–H). (**b**) Bacteria treated with AgNPs (rows A–D) and ZnO/TiO_2_/AgNCs (rows E–H).

To study the bacterial metabolic activity changes caused by the as-synthesised nanoparticles, a resazurin assay was performed, and the results are shown in Figure 6. The bacterial inhibitory effect was compared to that of bacteria without any treatment and blank MHB as a control. Figure 6a column 11 and Figure 6b column 10 represent the MHB control, and do not show any colour change. The untreated bacterial cells were metabolically active and appeared pink in colour due to the reduction of resazurin dye to resofurin showed in Figure 6a column 10 and Figure 6b column 9. In contrast, the cells treated with increasing dosage of nanoparticles showed a gradual rise in blue colour because of the deficiency of respiration, and are considered as inactive or dead bacteria. From the results in Table 1, the MIC of TiO_2_/AgNCs was found to be 31.2 μg/mL, and can minimally inhibit *E. coil, B. subtilis, P. aeruginosa*, while 62.5 μg/mL was needed to inhibit the growth of *S. aureus*. In the case of ZnO/TiO_2_/Ag, it demonstrated a higher inhibitory effect at the concentrations of 15.6 μg/mL and 31.2 μg/mL for *B. subtilis* and *P. aeruginosa* and *S. aureus* and *E. coli* respectively. The results revealed that the enhanced bactericidal activity recorded for the ZnO/TiO_2_/AgNCs are consistent with the MIC results.

#### 2.2.3. FESEM Analysis of Bacteria Morphological Changes

**Figure 7:**
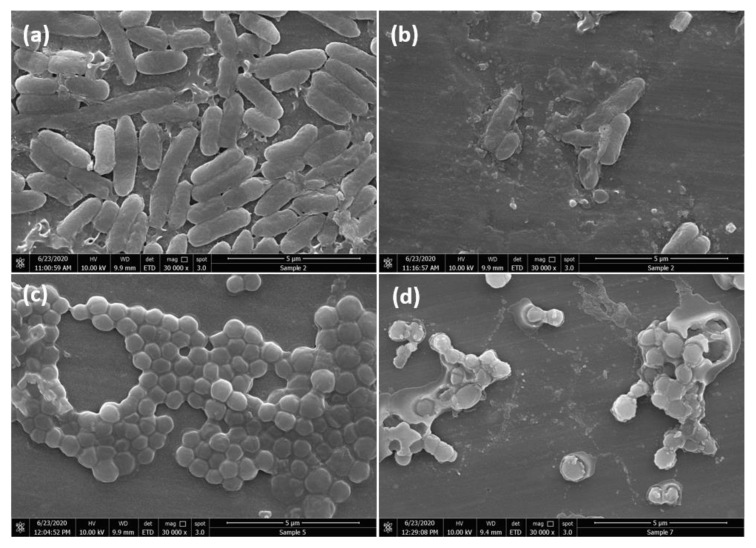
FESEM images of *E. coli* and *S. aureus* without any treatment, showing intact cell membrane (**a**,**c**), and the cells treated with ZnO/TiO_2_/AgNCs after 2 h, showing damaged cell membrane and leakage of the cellular contents (**b**,**d**).

The morphological changes and possible interactions with the bacteria *E. coli* and *S. aureus* caused by ZnO/TiO_2_/AgNCs were investigated by FESEM analysis. The results showed that the control bacteria without any treatment appeared to be smooth with intact morphology, indicating that the cells were viable and damage-free (Figure 7a,c). In contrast, nanoparticles treated cells showed rough cell membrane with holes and pits and excretion of intracellular contents (Figure 7b,d). Nanoparticles have the capability to penetrate bacteria due to their smaller size, precipitate on the cytoplasmic surface, thus leading to the loss of structural integrity and altering the cellular metabolic functions [34], such as the production of a high amount of ROS species that contain hydroxyl radicals, superoxide radicals and hydrogen peroxide, which in turn affect the protein, DNA, ATP synthesis and enzyme activities, finally result in cell death. Together, the combined effect of Ag, ZnO and TiO_2_ may contribute to the synergistic effect of bacterial pathogen inactivation.

#### 2.2.4. Confocal Visual Observation of Live/Dead Cells and ROS Generation

**Figure 8:**
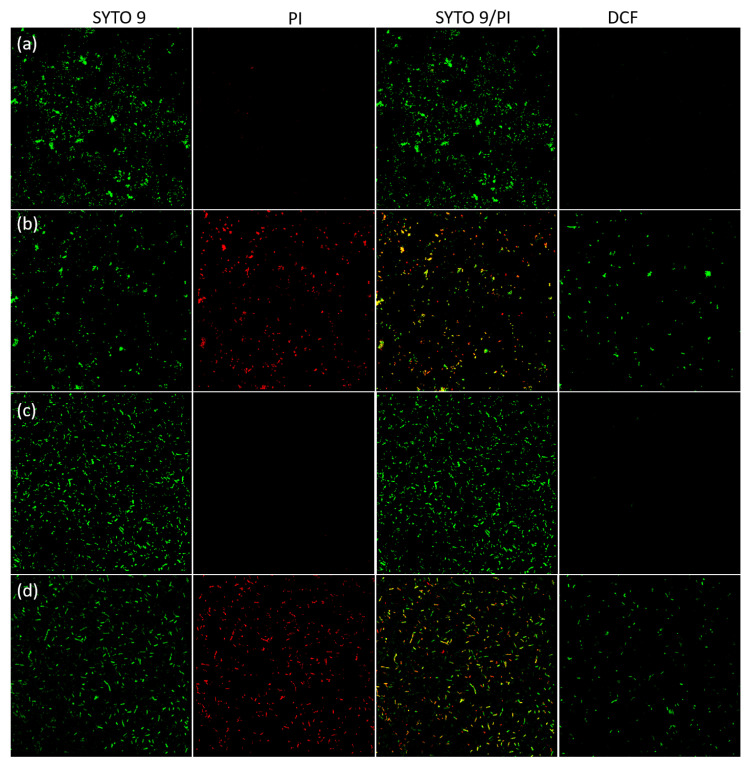
BacLight Viability assay (SYTO 9/PI) and ROS detection assay (DCF) CLSM images showing the *S. aureus* and *E. coli* (**a**,**c**) control and after treatment with ZnO/TiO_2_/AgNCs for 1 h (**b**,**d**). The merged images of SYTO 9/PI emit green fluorescence for viable cells while red:green indicates almost 60% of cell death. DCF emitted green fluorescent confirming the ROS generation on the treated cells (**b**,**d**) and no ROS were detected in control bacteria (**a**,**c**).

The bactericidal potential of ZnO/TiO_2_/AgNCs was determined by live/dead assay using SYTO 9, a green fluorescent dye that could bind all the viable and dead bacterial cells, and PI, a red fluorescent dye that specifically binds to the nucleic acids of damaged bacterial cells [35]. The bacterial cells of *S. aureus* and *E. coli* without any treatment were observed on the green and the green:red merged channels that emit a green colour, confirming that the cells were healthy and viable (Figure 8a,c). In contrast, the cells treated with ZnO/TiO_2_/AgNCs after 1 h showed green, orange and red colours, indicating that almost half of the total cells were exploited and compromised (Figure 8b,d). It has been reported that the bacterial surface in contact with nanocomposites resulted in the inactivation of the bacteria. Due to the strong interaction on the bacterial surface, the nanoparticles penetrate the bacterial membrane, altering the cellular respiration. Moreover, the high affinity of Ag to the thiol functional group of the protein leads to DNA condensation, which stops the DNA replication and, finally, results in bacterial cell death. A similar result of bacterial inhibition was observed in Ag and Zn incorporated TiO_2_ nanocomposites [36]. Most of the studies highlighting the bactericidal effect of Ag-based nanocomposites are controlled by the release of Ag^+^ ions to the bacterial population [37].

ROS generation was defined as a primary cause for antimicrobial activity of metal oxide nanostructures. Upon bacterial treatment with the nanocomposites, which induces the surface oxidative stress, the infused DCFDA is oxidised to DCF by using cellular ROS, which can be confirmed by confocal laser scanning microscope (CLSM). Figure 8 demonstrates the higher amount of ROS production in ZnO/TiO_2_/AgNCs exposed to *E. coli* and *S. aureus* by emitting the green fluorescence. On the contrary, the untreated control bacteria green fluorescent signal was not detected, showing that the cells were unaffected. A previous study reported that excessive production of ROS potentially alters the metabolic activity by damaging nucleic acids, protein synthesis and disintegrate the cell membrane [38]. The incorporation of ZnO and TiO_2_to the Ag could generate a high amount of hydrogen peroxide and hydroxyl radicals, which are destructive to the bacteria [39].

### 2.3. Cytotoxicity Analysis

#### 2.3.1. MTT Cell Viability

**Figure 9:**
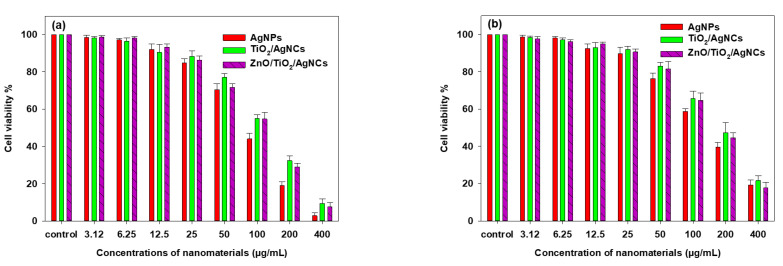
MTT cytotoxicity of AgNPs, TiO_2_/AgNCs and ZnO/TiO_2_/AgNCs against A549 lung cancer cells (**a**) and RAW264.7 macrophage cells (**b**).

To evaluate the biocompatibility of the as-synthesised antimicrobial materials, it is mandatory to study on their toxicity towards animal cells. The cytotoxicity effects of the as-synthesised AgNPs, TiO_2_/AgNCs and ZnO/TiO_2_/AgNCs were tested with RAW264.7 macrophage cell lines and A549 lung cancer cells. The results showed that AgNPs had displayed higher toxicity against A549 cells at the IC_50_ values of 95 µg/mL. In comparison, TiO_2_/AgNCs and ZnO/TiO_2_/AgNCs exhibited IC_50_ values of 130 µg/mL and 120 µg/mL, respectively (Figure 9a). Furthermore, cytotoxicity results of synthesised materials against RAW macrophages demonstrated higher biocompatibility at an IC_50_ dosage of 150 µg/mL for AgNPs and 200 µg/mL and 190 µg/mL for TiO_2_/AgNCs and ZnO/TiO_2_/AgNCs, respectively (Figure 9b). It is evident that the tested materials reduce the cell proliferation in a dose-dependent manner. Moreover, the TiO_2_/AgNCs and ZnO/TiO_2_/AgNCs expressed higher biocompatibility towards macrophages and expressed significant toxicity against lung cancer cells. This may be due to the high antioxidant property of healthy cells, which could tolerate the relative oxidative stress [40].

Ag has been known as a superior bactericidal agent from ancient times. A recent report suggested that nano-silver particles with an average size of 10 nm triggered the release of toxic Ag^+^ to human cells by damaging cellular organelles, metabolic changes and oxidative damage [41]. From the results, it is noted that for ZnO/TiO_2_/AgNCs, the IC_50_ dosage was significantly higher than that of AgNPs. Moreover, the dose-dependent toxicity of the resultant materials also correlates with the presence of Ag in the synthesised nanocomposites. Generally, Ag has been widely utilised for its potential biopharmaceutical properties compared to TiO_2_ and ZnO. However, our results suggested that the ternary composite material has remarkable antibacterial and anticancer properties without affecting normal human cells. In addition, medical grade alloys such as TiO_2_ and ZnO implants were already used for damaged bone [42]. In such conditions, the synthesised high biocompatible ZnO/TiO_2_/AgNCs could not only be used for bone tissue engineering applications, but also completely eradicate the bacterial pathogens on the target site. A previous study had reported that even lesser dosages of Ag or Ag-decorated nanocomposites are highly toxic to prokaryotic cells, while they do not show any significant cytotoxicity to eukaryotic cells due to their larger size and elevated structural redundancy over prokaryotes [33].

#### 2.3.2. Apoptosis

**Figure 10:**
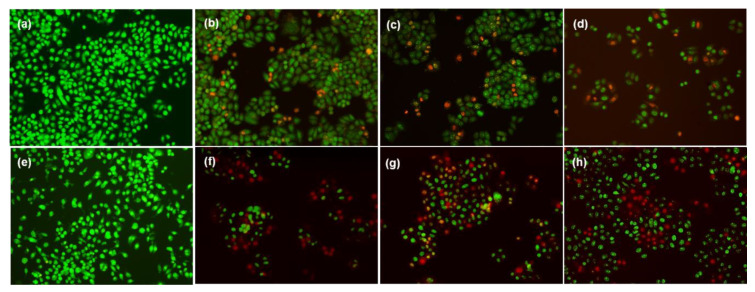
Fluorescence microscopic images of RAW macrophage cells control, A549 lung cancer cells control (**a**,**e**) and the cells exposed with AgNPs (**b**,**f**), TiO_2_/AgNCs (**c**,**g**) and ZnO/TiO_2_/AgNCs (**d**,**h**). Green fluorescence indicates viable cells; orange and red fluorescent colours indicate late apoptotic and dead cells.

Morphological changes of cells via the induction of apoptosis on A549 lung cancer cells and RAW 264.7 macrophage cell lines were qualitatively defined by AO/EB staining. AO, a membrane-permeable dye, can penetrate both viable and dead cells and has a green fluorescent colour, while EB, which penetrates only through the damaged cell membrane, emits a red colour. Figure 10b–d,f–h illustrate that all the nanoparticle-treated A549 lung cancer cells and RAW macrophage cells emitted green, orange and red colours, indicating signs of viable and apoptotic cells. Besides the IC_50_ dosage of the tested materials, ZnO/TiO_2_/AgNCs-exposed cells showed a higher percentage of apoptosis along with the necrosis than TiO_2_/AgNCs, and TiO_2_/AgNCs was higher than AgNPs. This may be because of the higher production of ROS generation, which is associated with the induction of early and late apoptosis towards killing the malignant cells [43]. Meanwhile, the cells without any treatment remained unaffected after 24 h (Figure 10a,e). A previous study revealed that apoptosis induced by AgNPs was regulated by the oxidative stress in lung cancer cell lines [44]. Reports have suggested that ROS generation is the major factor for triggering the apoptotic pathway [45] and that excessive production of ROS might devastate the antioxidant power of the cells, alter the cellular biochemical pathways and change the mitochondrial functions, leading to cell death [46]. Hence, this ZnO/TiO_2_/AgNCs could avail them as an ideal alternative in cancer therapeutic applications.

## 3. Conclusions

In this study AgNPs, TiO_2_NPs, binary TiO_2_/AgNCs and ternary ZnO/TiO_2_/AgNCs materials were synthesised via green chemistry procedures. The synthesised materials were characterised and evaluated for their antimicrobial potential and cytotoxicity. Specifically, the ternary nanocomposites showed optimal bactericidal effect towards *E. coli* and *S. aureus* and *P. aeruginosa* and *B. subtilis* at an MIC of 31.2 μg/mL and 15.6 μg/mL, respectively. The confocal and electron microscopic results inferred that the hybrid nanocomposite could potentially cause cell membrane disruption, altered biological functions and bacterial inactivation. The cytotoxicity results of AgNPs, TiO_2_/AgNCs and ZnO/TiO_2_/AgNCs against A549 and RAW264.7 macrophage cell lines expressed IC_50_ values of 95 µg/mL, 130 µg/mL and 120 µg/mL and 140 µg/mL, 200 µg/mL and 190 µg/mL respectively. The results of apoptosis showed that the hybrid nanocomposites expressed high toxicity towards cancer cells and lesser toxicity towards normal cells. Altogether, we conclude that the synthesised ZnO/TiO_2_/AgNCs has significantly inhibited the bacterial pathogens and cancer cells at lesser concentrations while showing higher biocompatibility to the normal cell lines. Hence, this study suggests that ZnO/TiO_2_/AgNCs could be used as a promising therapeutic antimicrobial.

## 4. Materials and Methods

### 4.1. Chemicals and Media Components

Silver nitrate (AgNO_3_), zinc acetate (Zn(CH_3_CO_2_)_2_ and titanium isopropoxide Ti[OCH(CH_3_)_2_]_4_ were purchased from Sigma Aldrich. Mueller Hinton broth and Mueller Hinton agar were purchased from Oxoid chemicals, UK. Roswell Park Memorial Institute (RPMI) 1640 medium and Penicillin Streptomycin antibiotics were purchased from Gibco, (Grand Island, New York NY, USA).

### 4.2. Plant Extract Preparation and Optimisation

Freshly ripened *Morinda citrifolia* fruit (MCF) was collected from the local market in Kuala Lumpur, Malaysia and washed with distilled water. Various concentrations of the extract were prepared by crushing different weights (4, 6, 8, 10 and 12 g) of MCF with 100 mL of MilliQ water. The extract was then microwave heated to boil and filtered using stainless steel wire mesh. Then the filtrate was centrifuged at 10,000 rpm for 8 min followed by filtering the supernatant using 0.22 µ vacuum filter (EMD Millipore TM stericup TM Sterile Vaccum driven filtration, Merck, Germany). The filtrate was stored at 4 °C for further use.

### 4.3. Nanoparticles Synthesis

**Figure 11:**
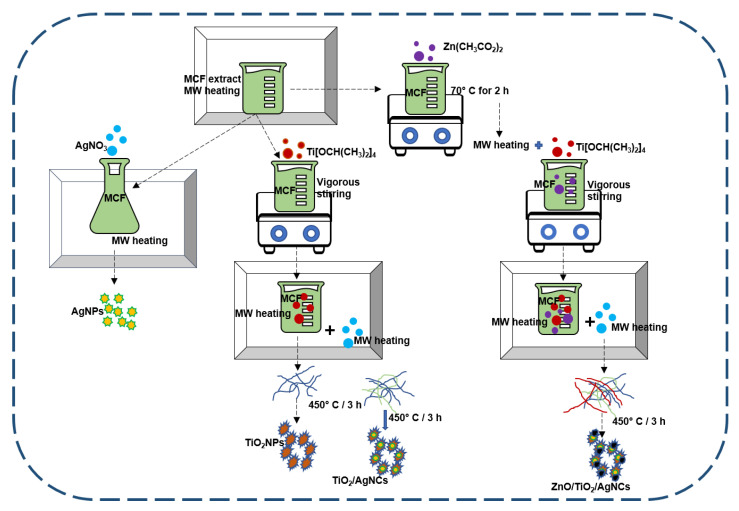
Schematic diagram of nanostructured materials synthesis using *M. citrifolia* extract via microwave-assistive techniques.

To identify the suitable MCF extract concentration for AgNPs synthesis, 100 mL of microwave preheated MCF extracts were separately added with a final volume concentration of 2 mM AgNO_3_ followed by microwave heating to observe the colour change. From the screening, 10 g of MCF in 100 mL water extract was selected as an ideal concentration for AgNPs synthesis. The AgNPs formation was visually confirmed by the colour change from a pale green colour reaction medium to a dark brown colour within 5 min. Then, the AgNP solution was cooled down to room temperature and collected by centrifugation at 13,000 rpm for 15 min. The obtained solid precipitate was washed three times using Milli-Q water to get pure AgNPs. A schematic diagram of the nanostructured materials synthesis is shown in Figure 11. Subsequently, the abovementioned MCF extract concentration was selected for all the nanoparticles synthesis. To synthesise TiO_2_NPs, 100 mL of microwave preheated MCF extract was mixed with dropwise addition of 500 µL Ti[OCH(CH_3_)_2_]_4_ under vigorous shaking using magnetic stirrer followed by microwave heating for 2 min. The solid titanium hydroxide precipitate was obtained, collected by centrifugation, washed and heated at 70 °C for 4 h. Then, the dried samples were calcined at 450 °C for 3 h to remove all the biological compounds. Finally, the white colour solid TiO_2_NPs was collected and stored for further studies.

To synthesise TiO_2_/AgNCs, titanium hydroxide precipitate was prepared according to the method mentioned above and added with a final volume concentration of 2 mM AgNO_3_, followed by microwave heating. The brownish yellow precipitate observed within a few minutes was collected, washed and dried at 70 °C for 4 h. Finally, TiO_2_/AgNCs were obtained by calcining the samples at 450 °C for 3 h. To synthesise the ternary ZnO/TiO_2_/AgNCs, 50 mM zinc acetate was added with 100 mL of MCF aqueous extract, and the samples were magnetically stirred at 70 °C. The reaction solution was subsequently added with NaOH solution until the zinc hydroxide precipitate was formed. After 2 h of continuous stirring, the reaction solution was added with 100 mL of microwave preheated MCF followed by dropwise addition of 1 mL Ti[OCH(CH_3_)_2_]_4_ under vigorous stirring followed by microwave heating. Then, the yellowish-brown resultant product was mixed with a final volume concentration of 3 mM AgNO_3_ and microwave heated for 5 min. The resultant brown final product was cooled down to room temperature, washed and dried, and the pure ZnO/TiO_2_/AgNCs were obtained after calcination.

### 4.4. Characterisation of Nanoparticles

The UV-visible absorbances of the MCF extract utilised for NPs synthesis and the aqueous NPs solution were scanned in the range of 200–700 nm at a wavelength of 1 nm using a Varioskan microplate reader. The FTIR analysis was performed for the MCF, resultant nanoparticles and nanocomposites using PerkinElmer Spectrum 400 instrument, USA, at a resolution of 4 cm^−1^ in the range of 650 to 4000 cm^−1^. The synthesised materials crystalline structure was determined by XRD using X’Pert Pro A analytical X-ray diffractometer instrument operated at 40 keV with Cu Ka and 2 theta from 20° to 80°. Nanoparticles shape and sizes were recorded using FESEM, JEOL, Japan. The sample preparation was carried out by loading a drop of diluted sample on aluminium foil, and vacuum dried for a day before viewing (Scanning Electron Microscopy (SEM) Quanta FEG 650) and Hitachi SU8220, Japan). The zeta potential of the synthesised materials was performed by using Malvern 130 Zetasizer Nano ZS90 instrument, UK.

### 4.5. Antimicrobial Activity of as-Synthesised Nanocomposites

#### 4.5.1. Determination of Minimum Inhibitory Concentration (MIC)

Antibacterial activities of TiO_2_, AgNPs, TiO_2_/Ag and ZnO/TiO_2_/AgNCs were examined against the nosocomial infection causing bacterial pathogens. Two Gram-negative and two Gram-positive strains such as *Pseudomonas aeruginosa* ATCC 27853 and *Escherichia coli* ATCC 25922 and *Staphylococcus aureus* NCTC 6571 and *Bacillus subtilis* ATCC 23857 were used in this study. The antibacterial assay was carried out following the standard microdilution method (clinical and laboratory standard institute, CLSI) with slight modifications. To determine the MIC, the selected bacterial strains were inoculated in Mueller Hinton Broth (MHB) and incubated at 180 rpm at 37 °C for 24 h. The diluted bacterial inoculum concentration 10^6^ colony forming unit (CFU/mL) was dispensed in 96-well plates containing various concentrations of TiO_2_NPs (1000, 500, 250, 125, 62.5, 31.25, 15.62, 7.8 and 3.9 µg/mL) through a two-fold dilution method and incubated at 37 °C for 24 h. Subsequently, the same method was followed to determine the MIC of AgNPs, TiO_2_/AgNCs and ZnO/TiO_2_/AgNCs with concentrations of 250, 125, 62.5, 31.25, 15.62, 7.8, 3.9 and 1.9 µg/mL, respectively. The bacterial inoculum treated with nanomaterials were incubated overnight at 37 °C. The bacteria without any treatment and blank MHB was used as a control, and optical density was measured at 600 nm [47].

#### 4.5.2. Resazurin Assay

The resazurin assay further determined the MIC of synthesised nanomaterials. The abovementioned MIC experimental condition was applied for this assay. The nanoparticles treated bacterial cells after the desired incubation, after which 20 µL of resazurin dye was incorporated to all the wells, which was incubated for 30 min at 37 °C to monitor the colour change. The MIC was detected based on the colour change from blue to a purple or pink colour [48].

#### 4.5.3. Bacterial Morphological Analysis upon Nanomaterials Exposure

The ZnO/TiO_2_/AgNCs interaction with the bacteria *E. coli* and *S. aureus* morphological changes were studied using FESEM. After a nanoparticle treatment for 2 h, the bacterial samples were washed with PBS, processed by glutaraldehyde fixation and dehydrated with serially diluted by ethanol and acetone and, finally, critical point dried samples were visualised under a FESEM (JEOL JSM7001F, Germany).

#### 4.5.4. Antibacterial Viability (Live/Dead Assay)

The ZnO/TiO_2_/AgNCs-exposed viable bacteria and dead cells were differentiated by using live/dead BacLight bacterial viability kit (Invitrogen, Carlsbad, CA, USA) containing SYTO 9 and propidium iodide (PI). Briefly, the nanocomposites-treated bacterial pathogens were kept in the shaker incubator for 1 h. Then, the bacteria pathogens were washed with PBS and resuspended with SYTO 9:Propidium Iodide (1:1) for 15 min. The samples were then loaded on the glass slide and viewed under CLSM (A Zeiss LSM 710-meta, Jena, Germany) with an excitation/emission wavelength of SYTO 9: 485/498 and PI: 535/617 with a 100× oil immersion objective lens.

#### 4.5.5. Detection of ROS Generation

The ROS production upon bacterial interaction with the synthesised nanomaterials was evaluated using DCFDA (a fluorescent probe, dichlorodihydrofluorescein diacetate) according to the manufacturer’s manual (DCFDA/H_2_DCFDA Cellular Reactive Oxygen Species Detection Assay Kit, Abcam, UK). The nonfluorescent DCFDA is converted to the highly fluorescent dichlorofluorescein (DCF) upon oxidation by ROS. Briefly, the freshly grown bacteria were treated with their MBC of synthesised nanomaterials and incubated for 4 h. Then, the cells were washed and stained with DCFDA for 30 min in the dark and viewed under CLSM. The bacteria without any treatment were related to the nanomaterials treated as a control.

### 4.6. Toxicity Assessment of Synthesised Materials

#### 4.6.1. MTT Assay

Synthesised AgNPs, TiO_2_/AgNCs and ZnO/TiO_2_/AgNCs MTT cytotoxicity, ROS generation and apoptosis induction were studied against A549 lung cancer cells and RAW264.7 macrophage cell lines. Roswell Park Memorial Institute (RPMI) 1640 medium complemented with 10% FBS under 5% CO_2_ at 37 °C. The experimental methods were followed according to the earlier report [49]. Finally, the absorbance was measured using a Varioskan microplate reader at a wavelength of 570 nm [50]. Cells without any treatment served as a control. The experiment was performed in triplicate, and the data were expressed as the mean of the three replicates in each experimental group ± SD.

#### 4.6.2. Detection of Apoptosis

AgNPs, TiO_2_/Ag and ZnO/TiO_2_/Ag nanocomposites’ IC_50_ dosage-exposed cells were incubated for 24 h, and apoptotic toxicity was detected by AO-EB dual staining. After treatment, the cells were washed with PBS and AO:EB (100 µg/mL) was added to all the wells. After 5 min, the cells were visualised under a fluorescence microscope (Olympus, BX-60, Tokyo, Japan).

## Figures and Tables

**Table 1:** MIC and Minimum Bactericidal Concentration (MBC) of TiO_2_NPs, AgNPs, TiO_2_/AgNCs and ZnO/TiO_2_/AgNCs, determined by broth microdilution assay against bacterial pathogens.

Bacterial Strains	TiO_2_NPs (µg/mL)		AgNPs (µg/mL)		TiO_2_/AgNCs (µg/mL)		ZnO/TiO_2_/AgNCs (µg/mL)	
	MIC	MBC	MIC	MBC	MIC	MBC	MIC	MBC
*E. coli*	500	500	31.2	31.2	31.2	31.2	31.2	31.2
*P. aeruginosa*	500	500	62.5	62.5	31.2	31.2	15.6	31.2
*S. aureus*	500	500	62.5	62.5	62.5	62.5	31.2	31.2
*B. subtilis*	250	250	31.2	62.5	31.2	62.5	15.6	15.6

## Data Availability

We have refered and agreed the MDPI Research Data Policies.
